# Association of Troponin T measurements with long-term outcomes in patients with coronary artery disease participating in a secondary prevention trial

**DOI:** 10.1186/s12872-023-03249-0

**Published:** 2023-04-28

**Authors:** Anete Kaldal, Serena Tonstad, Jarle Jortveit

**Affiliations:** 1 Department of Research, Sørlandet Hospital, Box 416 Lundsiden, 4604 Arendal, Norway; 2 Department of Endocrinology, Obesity and Preventive Medicine, Section of Preventive Cardiology, Oslo University Hospital, Oslo, Norway; 3 Department of Cardiology, Sørlandet Hospital, Arendal, Norway

**Keywords:** Cardiac troponin, Coronary heart diseases, Secondary prevention

## Abstract

**Background and aims:**

Identification of high-risk patients in secondary cardiovascular prevention may be challenging, although risk stratification tools are available. Cardiac troponins might have predictive value in identification of high-risk patients. The aim of this study was to investigate the association between cardiac Troponin T (cTnT) levels following a coronary event and long-term outcomes.

**Methods:**

This study was carried out as a subanalysis from a randomized controlled trial conducted at Sørlandet Hospital, Norway, where patients hospitalized with myocardial infarction (MI) or scheduled percutaneous coronary intervention (PCI)/coronary artery bypass grafting (CABG) were included between 2007 and 2017. Participants were followed-up for up to 10 years after the index event through out-patient consultations. cTnT was assessed at each consultation as well as information regarding new cardiovascular events or death.

**Results:**

A total of 1278 patients (18–80 years) with complete measurements of cTnT were included. cTnT was elevated (≥ 14 ng/L) one year after the primary event in 241 (19%) of participants. Median follow-up was 5.7 [SD 2.7] years. Cox regression analyses showed reduced survival (adjusted HR 0.37, 95% CI 0.19–0.72; *p* = 0.003) and composite endpoint-free survival (adjusted HR 0.73, 95% CI 0.55–0.98; *p* = 0.04) in participants with elevated cTnT versus participants with low cTnT after adjustment for risk factors at inclusion and randomization assignment.

**Conclusions:**

Assessment of cTnT after coronary heart events may help identify patients at high risk of poor outcomes and might contribute to more focused secondary preventive treatment.

**Trial registration:**

The study is registered in ClinicalTrials.gov (NCT00679237).

## Introduction

Many patients with coronary heart disease (CHD) experience repeated events [1]. The European Society of Cardiology (ESC) and American Heart Association (AHA)/American College of Cardiology (ACC) have issued detailed guidelines on secondary prevention of CHD [2, 3]. However, large studies have demonstrated a remaining gap between the guidelines and the achievement of recommended targets for secondary prevention [4–8]. In clinical practice, detection of high-risk patients remain challenging. Cardiovascular risk prediction tools for use after an event have been developed, but there is a lag in the implementation of these [9–11]. Probability calculation is based on gender, age, smoking status, time since first cardiovascular event, type(s) of atherosclerotic vascular disease, history of diabetes, systolic blood pressure, use of antithrombotic treatment, as well as levels of high sensitivity C-reactive protein, creatinine and cholesterol, among others [9]. As the secondary preventive treatment options are continuously increasing, easy accessible additional information that might promote individualization, evaluation and adjustment of secondary preventive treatment strategies during follow-up may be of clinical value, and might contribute to further refinement of prediction tools.

Cardiac troponins (cTn) are regulatory proteins that control the calcium-mediated interaction of actin and myosin in myocytes. cTn is convenient to measure in hospitals and in primary care. Myocyte necrosis and increased myocyte membrane permeability can lead to release of cTn and consequently elevated levels of cTn in blood plasma [12]. cTn is the preferred blood test in the diagnostic evaluation of acute myocardial infarction (MI), and an elevated value is required for the diagnosis [13]. However, cTn may also be elevated in patients with heart failure, pulmonary embolism, Takotsubo cardiomyopathy, myocarditis, arrhythmias and/or renal failure. Chronic elevated cTn might be associated with increased mortality [14, 15]. Guidelines do not recommend routine use of cTn in secondary preventive follow-up after coronary heart events, but cTn may have prognostic value and might improve selection of high-risk patients who would benefit of more intensified follow-up strategies and interventions [16–18].

The aim of this study was to investigate the association between elevated cardiac Troponin T (cTnT) and long-term outcomes in patients followed up with secondary preventive interventions after MI, percutaneous coronary intervention (PCI) and coronary artery bypass grafting (CABG).

## Methods

### Study design and study population

This study was carried out as a subanalysis from an open, randomized, controlled trial conducted at Sørlandet Hospital Arendal, Norway in the period 2007–2022. The main study was focused on comparison of secondary cardiovascular prevention within primary health care and within hospital-based follow-up, and primary outcomes were all-cause mortality and composite endpoint consisting of all-cause mortality, recurrence of non-fatal MI, new PCI/CABG, and non-fatal stroke. Secondary outcomes were achievement of secondary preventive treatment targets.

Consecutive patients, aged 18 to 80 years, admitted to the hospital between 2007 and 2017 with a diagnosis of MI or after scheduled PCI/CABG, were randomized to hospital-based follow-up or to follow-up within primary health care, and written informed consent was obtained from each participant. The exclusion criteria were as follows: lack of ability to cooperate, known alcohol- or drug-abuse, use of narcotics, pregnancy or breast-feeding, serious comorbidity with a life expectancy less than two years, or participation in other secondary prevention studies. Only patients with a minimum of 12 months of follow-up were included in the analysis. The trial was carried out in compliance with Declaration of Helsinki. The study design and main study results have been described previously [8].

### Intervention

Regular outpatient consultations conducted by trained nurses were offered to patients randomized to the hospital-based follow-up group at two weeks, three months, six months, 12 months and thereafter annually for up to five years after the index event. The final data collection was completed after 10 years. The attainment of treatment targets was the focus throughout entire follow-up period. Blood pressure, weight, height, waist circumference, cTnT, LDL-cholesterol and HbA_1c_ were assessed at each consultation. Smoking status, diet, physical activity, and use of medication were reported by the participant. At each consecutive consultation data about death, hospital admissions, stroke, recurrent MI, or new PCI/CABG were recorded.

For patients randomized to the primary care group, follow-up with respect to the secondary preventive measures was conducted by the family physician. Advice regarding treatment targets was sent to the family physician when the patient was discharged from hospital after the index event. Study data in the primary care group was obtained through regular outpatient consultations at 12 months, two years, and five years with a final data collection at ten years after the index event. Identical study data as in the hospital-based follow-up group was collected through outpatient consultations, but without intervening in the treatment regimes.

#### Treatment targets of secondary prevention

The secondary preventive treatment targets were those recommended by the latest ESC guidelines available at the time of the study and included the following [2, 19–23]:

- No smoking
- Blood pressure < 140/90 mmHg
- LDL-cholesterol < 1.8 mmol/l (< 2.5 mmol/l until 2017, < 1.4 mmol/l from 2020)
- HbA_1c_ < 53 mmol/mol (7%)
- BMI < 25 kg/m^2^
- Daily use of lipid lowering therapy
- Daily use of acetylsalicylic acid
- Physical activity of moderate intensity for ≥ 150 min weekly

### Cardiac troponin

The serum concentration of cTnT was analyzed at the hospitals core lab (Roche Diagnostics). A cut of value of ≥ 14 ng/L (99th percentile) was chosen as the limit for an elevated value, and values below it were defined as low.

### Outcomes

The main outcome was composite endpoint-free survival based on all-cause mortality, PCI, CABG, non-fatal stroke, or non-fatal MI during the follow-up, in patients with or without elevated cTnT values one year after the index coronary event. Although cTnT was measured at each outpatient consultation, one-year measurements of cTnT were the first available measurements for both study groups and therefore chosen for analysis of main outcome.

### Statistical analysis

Continuous variables are reported as means ± SD (standard deviations) and differences between groups were analysed using independent samples t-tests. Categorical variables are presented as numbers and percentages, and differences between groups were analysed by the chi-squared test. Missing values are reported, and categorical variables are reported as proportion of non-missing values. Kaplan–Meier curve for composite endpoint-free survival and survival after hospital admission for the first MI or PCI/CABG in the study period was estimated. Cox regression analyses were used to calculate hazard ratios (HRs) with 95% confidence intervals (CIs). The regression analyses were adjusted for differences in established risk characteristics (age, gender, renal failure, type of index event, smoking, hypertension, diabetes, and previous MI, use of acetylsalicylic acid at 12 months) and randomization to hospital versus primary care follow-up. A *p*-value of < 0.05 was regarded as statistically significant. The analysis was not adjusted for multiple testing. The statistical analyses were performed by using STATA, version 17 (StataCorp, 4905 Lakeway Dr, College Station, TX 77,845, USA).

## Results

**Fig. 1:**
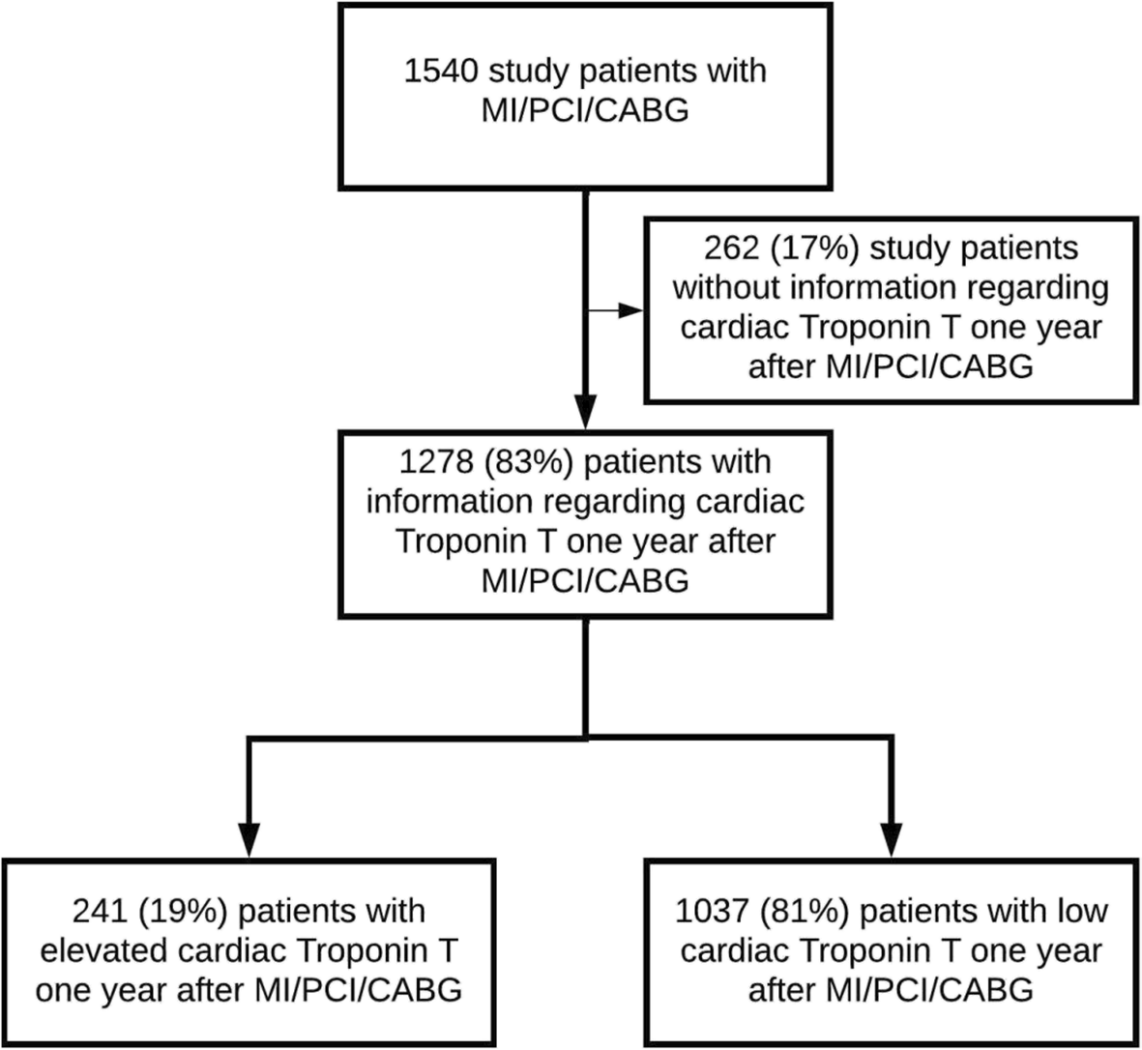
Study flow chart

A total of 1540 patients were eligible for inclusion (Fig. 1). Patients with missing cTnT values one year after the index event (*n* = 262 (17%)) were excluded from further analysis. Among the remaining 1278 patients, cTnT was elevated one year after the index event in 241 (19%) patients, with a median value of 21 ng/L (interquartile range 17–29). Most patients with elevated cTnT one year after index event, had elevated cTnT also after two (80%) and five years (79%) with stable median cTnT value. Among those without elevated values one year after the index event, 8% and 15% developed raised cTnT levels at two- and five-years follow-up, respectively. More patients in the hospital-based follow-up group had elevated cTnT compared to the primary care follow-up group (135 (21%) patients vs. 106 (16%) patients, *p* = 0.02) one year after the index event.

### Clinical characteristics

Baseline characteristics of patients with and without elevated cTnT are described in Table 1. Half of the patients included in the analysis (*n* = 604 (48%)) were hospitalized due to acute MI. Nearly equal proportions of the patients were followed-up at hospital (*n* = 631 (49%)) and in primary care (*n* = 647 (51%)). The patients with elevated cTnT values were older than the patients with low cTnT values. More men than women had elevated cTnT, while smokers had lower cTnT. Among baseline characteristics associated with elevated cTnT one year after the index event were hypertension, and lower LDL-cholesterol, diabetes, previous MI, and renal failure.

**Table 1:** Baseline clinical characteristics at hospitalization for acute myocardial infarction (MI), percutaneous coronary intervention (PCI) or coronary artery bypass grafting (CABG) in patients with and without elevated cardiac Troponin T (< 14 ng/L) one year after the index event

	Missing values	**Elevated cardiac Troponin T**	**Low cardiac Troponin T**	***p***
	*n* = 1278	*n* = 241	*n* = 1037	
Mean age (years) (SD)	0 (0)	69 (9)	62 (8)	< 0.001
Men (%)	0 (0)	207 (86)	800 (77)	0.003
Mean body mass index (kg/m2) (SD)	137 (11)	29 (5)	28 (4)	< 0.001
Smoking (%)	7 (0)	48 (20)	278 (27)	0.03
Lipid lowering therapy (%)	40 (3)	126 (56)	462 (46)	0.007
Antihypertensive therapy (%)	23 (2)	147 (64)	470 (46)	< 0.001
Diabetes (%)	3 (0)	68 (28)	132 (13)	< 0.001
Previous coronary heart disease:				
*Previous myocardial infarction (%)*	7 (0)	49 (21)	125 (12)	0.001
*Previous percutaneous coronary intervention (emergency or scheduled) (%)*	1 (0)	35 (15)	140 (14)	0.68
*Previous coronary artery bypass grafting (emergency or scheduled) (%)*	2 (0)	18 (7)	53 (5)	0.15
Renal failure	9 (1)	20 (8)	13 (1)	< 0.001
Mean LDL-cholesterol (mmol/L) (SD)	41 (3)	2.6 (1.0)	3.0 (1.1)	< 0.001
Mean systolic blood pressure (mmHg) (SD)	2 (0)	148 (24)	147 (24)	0.29
Mean diastolic blood pressure (mmHg) (SD)	2 (0)	86 (15)	87 (14)	0.16
Acute myocardial infarction as qualifying diagnosis (%)	16 (1)	97 (41)	507 (50)	0.02

Patients with elevated cTnT achieved on average 4.1 [SD 1.2] of the seven general treatment targets (excluding HbA1c in patients with diabetes) for secondary prevention while patients with low cTnT achieved 4.3 [SD 1.1] treatment targets (*p* = 0.01) within one year after MI, PCI or CABG. Achievement of different treatment targets for secondary prevention is presented in Table 2.

**Table 2:** Secondary preventive target achievement for cardiovascular risk factors and medication use in patients with and without elevated cardiac Troponin T (< 14 ng/L) one year after myocardial infarction/percutaneous coronary intervention/coronary artery bypass grafting

**Target achievement, (%)**^**a**^	Missing values	**Elevated cardiac Troponin T**	**Low cardiac Troponin T**	***p***
	*n* = 1278	*n* = 241	*n* = 1037	
Non-smoking	6 (0)	208 (86)	825 (80)	0.02
Blood pressure	3 (0)	131 (54)	621 (60)	0.11
LDL-cholesterol	36 (3)	186 (77)	722 (72)	0.11
HbA1c (if diabetes, *n* = 195)	10 (5)	26 (41)	70 (57)	0.04
Body mass index	90 (7)	49 (22)	235 (24)	0.48
Lipid lowering therapy	18 (1)	229 (96)	972 (95)	0.69
Acetylsalicylic acid	7 (1)	216 (90)	991 (96)	< 0.001
Physical activity	0 (0)	130 (54)	621 (60)	0.09

^a^non-smoking, blood pressure < 140/90 mmHg, LDL-cholesterol < 2.5 mmol/l (2007–2017)/ < 1.8 mmol/l (2018–20,200)/ < 1.4 mmol/l (2021-), HbA1c < 53 mmol/l (7%), body mass index < 25 kg/m2, daily use of lipid lowering therapy, daily use of acetylsalicylic acid, physical activity of minimum moderate intensity ≥ 150 min weekly

### Outcomes

**Fig. 2:**
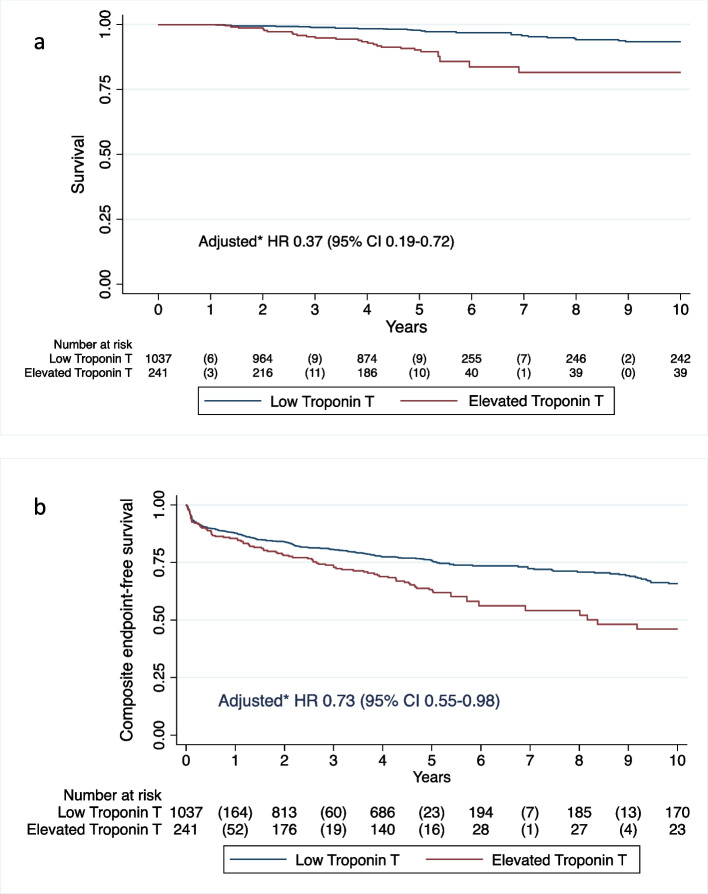
**a** Survival in patients with and without elevated cardiac troponin T value one year after myocardial infarction (MI), percutaneous coronary intervention (PCI) or coronary artery bypass grafting (CABG). **b** Composite endpoint-free survival in patients with and without elevated cardiac troponin T value one year after myocardial infarction (MI), percutaneous coronary intervention (PCI) or coronary artery bypass grafting (CABG). *Adjusted for following baseline characteristics: age, sex, randomization group, smoking, presence of renal failure, diabetes, hypertension, history of MI, use of acetylsalicylic acid at 12 months, and qualifying diagnosis (MI or planned PCI/CABG)

After a median follow-up time of 5.7 [SD 2.7] years, patients with low cTnT had lower all-cause mortality than patients with elevated cTnT (adjusted HR 0.37, 95% CI 0.19–0.72; *p* = 0.003) (Fig. 2a). Similar, composite endpoint-free survival in patients with elevated cTnT one year after MI, PCI or CABG was reduced compared to patients with low cTnT (adjusted HR 0.73, 95% CI 0.55–0.98; *p* = 0.04) (Table 3 and Fig. 2b).

**Table 3:** Composite endpoint (all-cause death, non-fatal myocardial infarction (MI), percutaneous coronary intervention (PCI), coronary artery bypass grafting (CABG) or non-fatal stroke) and total number of cardiovascular events in patients with and without elevated cardiac Troponin T (< 14 ng/L) one year after myocardial infarction/percutaneous coronary intervention/coronary artery bypass grafting

	**Elevated cardiac Troponin T**	**Low cardiac Troponin T**	p*
	*n* = 241	*n* = 1037	
**Composite endpoint**	93 (38.6)	268 (25.8)	< 0.001
Death (%)	25 (10.4)	33 (3.2)	< 0.001
Myocardial infarction (%)	16 (6.6)	47 (4.5)	0.18
Percutaneous coronary intervention (%)	57 (23.7)	205 (19.8)	0.18
Coronary artery bypass grafting (%)	5 (2.1)	8 (0.8)	0.07
Stroke (%)	18 (7.5)	32 (3.1)	0.002

Mean follow-up time: 5.7 (SD 2.7) years

^*^p-values referring to differences in proportions between group with or without elevated Troponin T

## Discussion

Patients with elevated cTnT one year after MI, PCI or CABG had decreased long-term composite endpoint-free and crude survival compared to patients with low cTnT values and achieved slightly less secondary preventive targets for cardiovascular risk factors.

About 30% of all participants experienced a new major cardiovascular event (MACE) during the median follow-up period of 5.7 years. Elevated cTnT was associated to poorer outcome. Cardiac TnT is reported from other studies to be a robust predictor of mortality [24–26]. This reiterates the importance of improving secondary preventive treatment after MI, PCI and/or CABG for patients with chronic elevated cTnT. In our study, cTnT was the routine assessment at the index event and during the follow-up, and cardiac troponin I (cTnI) was not measured. Recent data points to prognostic differences between these cardiac markers: cTnT has higher predictive value with regard to cardiovascular and all-cause mortality, while cTnI is better indicator for ischemic events and coronary artery disease [27, 28]. Our data must therefore be interpreted in the light of these differences, as we observed higher mortality among patients with elevated cTnT, while MI, PCI or CABG did not differ significantly. cTnT elevation after ischemic stroke or TIA has been described as a predictor for recurrent cardiovascular events, including stroke, and mortality [29, 30]. Our data support recently described reciprocal association with elevated cTnT predicting stroke [31].

The prevalence of elevated cTnT (19%) in our study population of patients with chronic stable coronary heart disease was comparable with previous studies [24, 32, 33]. The underlying mechanisms for elevated cTnT are unclear, but elevated cTnT is shown to be independently associated with e.g. age, gender, BMI and LV function [24]. Significantly greater proportion of non-smokers had elevated cTnT. Previous study has described lower cTnT values among smokers [34]. The underlying mechanisms are not well understood, and the prognostic value of cTnT levels in smokers is less clear [34]. Ongoing acetylsalicylic acid (ASA) treatment have shown to be a significant predictor for lower cTnT levels in acute myocardial infarction, and intracoronary infusion of ASA seem to reduce oxidative stress in ischemia–reperfusion setting [35, 36]. We found significantly lower proportion of participants on ASA treatment among those with elevated cTnT levels. Although recently questioned, lifelong treatment with acetylsalicylic acid in secondary prevention has been well established practice for four decades [37]. Whether results from our study may indicate the preventive value of ASA beyond antithrombotic properties, or rather reflects differences between patients with or without this therapy, requires separate investigation.

Patients with elevated cTnT reached slightly less targets for secondary prevention than patients with low cTnT values. Although the results cannot prove causal relations between achievement of the treatment targets and cTnT values, the study revealed a potential for improvement in the overall rate of treatment target achievement for all patients. 30–50% of the participants did not reach the treatment targets for blood-pressure and LDL-cholesterol. Most of the patients did not achieve treatment targets for the lifestyle factors body mass index, smoking, and physical activity. We find reason to stress the importance of adherence to the secondary preventive guideline from ESC, AHA and ACC for all patients.

More patients in the hospital-based follow-up group had elevated cTnT compared to the primary care follow-up group. Taking in account the observational value of the results in this subanalysis, we may just suppose some possible explanations. One of these might be that patients in hospital follow-up (intervention) group were more compliant to the follow-up across the whole group, while in primary care follow-up group patients with multiple comorbidities and more complex medical history dropped the out-patient consultations in greater extent due to comorbidities itself or concurring appointments. In the latter group out-patient consultations included only data collection without intervention, thus probably patients with greater morbidity and more frequent appointment burden would prioritize those appointments with greater clinical relevance.

In our opinion, the study results indicate a potential usefulness of cTnT in secondary preventive follow-up after MI, PCI and/or CABG. Measurements of cTnT identify patients at high risk who may benefit from further optimalization of secondary preventive therapy.

This study was limited to one hospital and a limited number of endpoints. Smoking status, amount of exercise and use of medications were self-reported, and likely to be affected by reporting bias. Furthermore, we lacked information regarding left ventricular systolic function in most patients. Nevertheless, this study included patients from both hospital-based and primary care based secondary preventive follow-up and represents a real-life population of patients with stable coronary heart disease.

## Conclusion

Patients with elevated cTnT one year after MI, PCI and/or CABG had decreased composite endpoint-free and crude survival, and a smaller proportion in this group were on ASA treatment. Assessment of Troponin T in secondary preventive follow-up after coronary heart events might identify patients at high risk and might contribute to optimizing of the secondary preventive treatment, as well as further refinement of cardiovascular risk prediction tools.

## Data Availability

The study protocol and data are available on request due to privacy/ethical restrictions, the corresponding author should be contacted in case.

## Abbreviations

BMI: Body mass index
CABG: Coronary artery bypass grafting
CVD: Cardiovascular diseases
HbA_1c_: Glycated hemoglobin
LDL: Low-density lipoprotein
MI: Myocardial infarction
PCI: Percutaneous coronary intervention
cTn: Cardiac Troponin
cTnT: Cardiac Troponin T

## References

[1] Jortveit J, Halvorsen S, Kaldal A (2019). Unsatisfactory risk factor control and high rate of new cardiovascular events in patients with myocardial infarction and prior coronary artery disease. BMC Cardiovasc Disord.

[2] Piepoli MF, Hoes AW, Agewall S (2016). 2016 European Guidelines on cardiovascular disease prevention in clinical practice: The Sixth Joint Task Force of the European Society of Cardiology and Other Societies on Cardiovascular Disease Prevention in Clinical Practice (constituted by representatives of 10 societies and by invited experts)Developed with the special contribution of the European Association for Cardiovascular Prevention & Rehabilitation (EACPR). Eur Heart J.

[3] Smith SC, Benjamin EJ, Bonow RO (2011). AHA/ACCF Secondary Prevention and Risk Reduction Therapy for Patients With Coronary and Other Atherosclerotic Vascular Disease: 2011 Update. J Am Coll Cardiol.

[4] Kotseva K, Wood D, De Backer G (2009). Cardiovascular prevention guidelines in daily practice: a comparison of EUROASPIRE I, II, and III surveys in eight European countries. Lancet.

[5] Kotseva K, Wood D, De Bacquer D (2016). EUROASPIRE IV: A European Society of Cardiology survey on the lifestyle, risk factor and therapeutic management of coronary patients from 24 European countries. Eur J Prev Cardiol.

[6] Ferrari R, Ford I, Greenlaw N (2014). Geographical variations in the prevalence and management of cardiovascular risk factors in outpatients with CAD: Data from the contemporary CLARIFY registry. Eur J Prev Cardiol.

[7] Cacoub PP, Zeymer U, Limbourg T (2011). Effects of adherence to guidelines for the control of major cardiovascular risk factors on outcomes in the REduction of Atherothrombosis for Continued Health (REACH) Registry Europe. Heart.

[8] Kaldal A, Tonstad S, Jortveit J (2021). Long-term hospital-based secondary prevention of coronary artery disease: a randomized controlled trial. BMC Cardiovasc Disord.

[9] Jacobs JJ, Hutter C, Tabak F, et al. https://u-prevent.com/calculators (Accessed 25.08 2022).

[10] Muthee TB, Kimathi D, Richards GC (2020). Factors influencing the implementation of cardiovascular risk scoring in primary care: a mixed-method systematic review. Implement Sci.

[11] World Health Organization. Assessing national capacity for the prevention and control of non-communicable diseases: report of the 2015 global survey, https://apps.who.int/iris/bitstream/handle/10665/246223/9789241565363-eng.pdf?sequence=1 (2015, Accessed 15.07.22).

[12] Higgins JP, Higgins JA (2003). Elevation of cardiac troponin I indicates more than myocardial ischemia. Clin Invest Med.

[13] Thygesen K, Alpert JS, Jaffe AS, et al. Fourth universal definition of myocardial infarction (2018). Eur Heart J 2018 2018/08/31. DOI: 10.1093/eurheartj/ehy462. 10.1016/j.gheart.2018.08.004 30154043

[14] Saunders JT, Nambi V, de Lemos JA (2011). Cardiac troponin T measured by a highly sensitive assay predicts coronary heart disease, heart failure, and mortality in the Atherosclerosis Risk in Communities Study. Circulation.

[15] de Lemos JA, Drazner MH, Omland T (2010). Association of troponin T detected with a highly sensitive assay and cardiac structure and mortality risk in the general population. JAMA.

[16] Visseren FLJ, Mach F, Smulders YM (2021). 2021 ESC Guidelines on cardiovascular disease prevention in clinical practice. Eur Heart J.

[17] White HD, Tonkin A, Simes J (2014). Association of contemporary sensitive troponin I levels at baseline and change at 1 year with long-term coronary events following myocardial infarction or unstable angina: results from the LIPID Study (Long-Term Intervention With Pravastatin in Ischaemic Disease). J Am Coll Cardiol.

[18] Marston NA, Bonaca MP, Jarolim P (2020). Clinical Application of High-Sensitivity Troponin Testing in the Atherosclerotic Cardiovascular Disease Framework of the Current Cholesterol Guidelines. JAMA Cardiol.

[19] Graham I, Atar D, Borch-Johnsen K (2007). European guidelines on cardiovascular disease prevention in clinical practice: executive summary: Fourth Joint Task Force of the European Society of Cardiology and Other Societies on Cardiovascular Disease Prevention in Clinical Practice (Constituted by representatives of nine societies and by invited experts). Eur Heart J.

[20] Perk J, De Backer G, Gohlke H, Guidelines European, on cardiovascular disease prevention in clinical practice (version, (2012). The Fifth Joint Task Force of the European Society of Cardiology and Other Societies on Cardiovascular Disease Prevention in Clinical Practice (constituted by representatives of nine societies and by invited experts). Eur Heart J.

[21] Ibanez B, James S, Agewall S (2017). 2017 ESC Guidelines for the management of acute myocardial infarction in patients presenting with ST-segment elevation: The Task Force for the management of acute myocardial infarction in patients presenting with ST-segment elevation of the European Society of Cardiology (ESC). Eur Heart J.

[22] Mach F, Baigent C, Catapano AL (2019). 2019 ESC/EAS Guidelines for the management of dyslipidaemias: lipid modification to reduce cardiovascular risk: The Task Force for the management of dyslipidaemias of the European Society of Cardiology (ESC) and European Atherosclerosis Society (EAS). Eur Heart J.

[23] Collet J-P, Thiele H, Barbato E, et al. 2020 ESC Guidelines for the management of acute coronary syndromes in patients presenting without persistent ST-segment elevation: The Task Force for the management of acute coronary syndromes in patients presenting without persistent ST-segment elevation of the European Society of Cardiology (ESC). Eur Heart J 2020. DOI: 10.1093/eurheartj/ehaa575. 10.1093/eurheartj/ehaa624 33085966

[24] Biener M, Giannitsis E, Lamerz J (2016). Prognostic value of elevated high-sensitivity cardiac troponin T levels in a low risk outpatient population with cardiovascular disease. Eur Heart J Acute Cardiovasc Care.

[25] Mueller M, Celik S, Biener M (2012). Diagnostic and prognostic performance of a novel high-sensitivity cardiac troponin T assay compared to a contemporary sensitive cardiac troponin I assay in patients with acute coronary syndrome. Clin Res Cardiol.

[26] Celik S, Giannitsis E, Wollert KC (2011). Cardiac troponin T concentrations above the 99th percentile value as measured by a new high-sensitivity assay predict long-term prognosis in patients with acute coronary syndromes undergoing routine early invasive strategy. Clin Res Cardiol.

[27] Árnadóttir Á, Vestergaard KR, Pallisgaard J (2018). High-sensitivity cardiac troponin T is superior to troponin I in the prediction of mortality in patients without acute coronary syndrome. Int J Cardiol.

[28] Eggers KM, Hammarsten O and Lindahl B. Differences between high-sensitivity cardiac troponin T and I in stable populations: underlying causes and clinical implications. Clin Chem Lab Med 2022 2022/11/26. DOI: 10.1515/cclm-2022-0778. 10.1515/cclm-2022-0778 36424851

[29] Broersen LHA, Stengl H, Nolte CH (2020). Association Between High-Sensitivity Cardiac Troponin and Risk of Stroke in 96 702 Individuals. Stroke.

[30] Hellwig S, Ihl T, Ganeshan R (2021). Cardiac Troponin and Recurrent Major Vascular Events after Minor Stroke or Transient Ischemic Attack. Ann Neurol.

[31] Kobayashi T, Nasu T, Satoh M (2022). Association between high-sensitivity cardiac troponin T levels and incident stroke in the elderly Japanese population: Results from the Tohoku Medical Megabank Community-based Cohort Study. American Heart Journal Plus: Cardiology Research and Practice.

[32] Beatty AL, Ku IA, Christenson RH (2013). High-sensitivity cardiac troponin T levels and secondary events in outpatients with coronary heart disease from the Heart and Soul Study. JAMA Intern Med.

[33] Koenig W, Breitling LP, Hahmann H (2012). Cardiac troponin T measured by a high-sensitivity assay predicts recurrent cardiovascular events in stable coronary heart disease patients with 8-year follow-up. Clin Chem.

[34] Skranes JB, Claggett BL, Myhre PL (2019). Current Smoking Is Associated With Lower Concentrations of High-Sensitivity Cardiac Troponin T in Patients With Stable Coronary Artery Disease. Circulation.

[35] Bhatt HA, Sanghani DR, Lee D (2016). Predictors of Peak Troponin Level in Acute Coronary Syndromes: Prior Aspirin Use and SYNTAX Score. Int J Angiol.

[36] Frydrychowski P, Michałek M, Bil-Lula I (2022). Cardioprotective Effect of Acetylsalicylic Acid in the Myocardial Ischemia-Reperfusion Model on Oxidative Stress Markers Levels in Heart Muscle and Serum. Antioxidants.

[37] Jacobsen AP, Raber I, McCarthy CP (2020). Lifelong Aspirin for All in the Secondary Prevention of Chronic Coronary Syndrome. Circulation.

